# Validity and reliability of the Thai version of the simple frailty questionnaire (T-FRAIL) with modifications to improve its diagnostic properties in the preoperative setting

**DOI:** 10.1186/s12877-022-02863-5

**Published:** 2022-02-28

**Authors:** Warut T.Sriwong, Waroonkarn Mahavisessin, Varalak Srinonprasert, Arunotai Siriussawakul, Wichai Aekplakorn, Panita Limpawattana, Patumporn Suraarunsumrit, Rachaneekorn Ramlee, Titima Wongviriyawong

**Affiliations:** 1 Department of Medicine, Faculty of Medicine Khon Kaen University, Khon Kaen, 40002 Thailand; 2 Department of Anesthesiology, Faculty of Medicine Khon Kaen University, Khon Kaen, 40002 Thailand; 3 Division of Geriatric Medicine, Department of Medicine, Faculty of Medicine Siriraj Hospital, Mahidol University, Bangkok, 10700 Thailand; 4 Integrated Perioperative Geriatric Excellent Research Center, Faculty of Medicine, Siriraj Hospital, Mahidol University, Bangkok, 10700 Thailand; 5 Department of Anesthesiology, Faculty of Medicine Siriraj Hospital, Mahidol University, Bangkok, 10700 Thailand; 6 Department of Community Medicine, Faculty of Medicine Ramathibodi Hospital, Mahidol University, Bangkok, 10400 Thailand; 7 Division of Geriatric Medicine, Department of Medicine, Faculty of Medicine Khon Kaen University, Khon Kaen, 40002 Thailand

**Keywords:** Frailty, Elderly patients, Diagnosis, Translation, Validity, Reliability

## Abstract

**Background:**

Several methods are available for identifying frailty, but limited tools have been validated in Thai context. Our objective was to evaluate the validity and reliability of the Thai version of the Simple Frailty Questionnaire (T- FRAIL) compared to the Thai Frailty Index (TFI) and to explore modifications to improve its diagnostic properties.

**Methods:**

The T-FRAIL was translated with permission using a standardized protocol, that included forward and back-translation. Content validity analysis was performed using input from 5 geriatricians. Test-retest reliability, concurrent validity, diagnostic properties, and options to increase the sensitivity of the questionnaire were explored. A cross-sectional study for evaluation validity and reliability was carried out among 3 hundred patients aged 60 or more undergoing elective surgery at a university hospital.

**Results:**

The item content validity index (I-CVI) showed 1.0 for each questionnaire item. Test-retest reliability within a 7-day interval was done in 30 patients with a good intraclass correlation coefficient of 0.880. Compared with the TFI, the T-FRAIL yielded an excellent accuracy (area under the curve = 0.882). The identification of frailty using a score of 2 points or more provided the best Youden’s index at 63.1 with a sensitivity of 77.5% (95% CI 69.0–84.6) and a specificity of 85.6% (95% CI 79.6–90.3). A cutoff point of 1 out of 5 items for original T-FRAIL provided a sensitivity of 93.3% and a specificity of 61.1%. The modified T-FRAIL (T-FRAIL_M1), by reducing the “illnesses” criterion to 4 or more diseases, at a cutoff point at 1 had a sensitivity of 94.2% and a specificity of 57.8%. Another modified T-FRAIL (T-FRAIL_M2), by combining three components, at a cutoff point at 1 yielded a sensitivity of 85.8% and a specificity of 80.6%.

**Conclusion:**

The T-FRAIL and its modification demonstrated satisfactory validity and reliability to identify frailty in elderly patients. The cutoff score of 1 point from 5 items from the original version of T-FRAIL and T-FRAIL_M1 provides a highly sensitive screening tool. T-FRAIL_M1 with a cutoff point of 2 and T-FRAIL_M2 yields reasonable sensitivity and specificity for practical use.

## Background

Frailty is a clinical syndrome characterized by reduced physiological body reserve that was originally described in older persons [1, 2], but is increasingly identified in younger age groups [3–5]. This vulnerable state results in decreased responses to internal and external stressors that lead to negative clinical consequences [6]. Frailty has been associated with a higher number of comorbidities, increased incidence of falls, and more disability in older people [7–9]. Increased rates of hospital admission and mortality have been observed in studies of frailty [10, 11].

Frailty screening reliably classifies high-risk older patients in several medical contexts, including surgical care [12–14]. Frail patients are more likely to experience negative postoperative consequences [15, 16]. One systematic review reported a strong relationship between frailty and major adverse cardiac events after cardiac surgery [17]. In cancer patients, frailty status predicts outcomes for many modalities of treatments, including patients undergoing surgical intervention [18].

Improved methods to identify frailty in older, surgical patients could lead to better perioperative care. Preoperative preparation including prehabilitation, correcting malnutrition, psychological intervention, and prevention of postoperative delirium have been recommended to improve the quality of care. Shared decision-making is also suggested to better prepare frail patients for surgery [19, 20]. Intraoperative management of altered pharmacodynamic and pharmacokinetics [21], and postoperative multidisciplinary care in frail patients are also beneficial [22]. Therefore, frailty is included in the list for optimal preoperative assessment of older surgical patients guideline recommended by the American College of Surgeons National Surgical Quality Improvement Program and the American Geriatrics Society [23].

Several approaches have been proposed to identify frailty [2, 24–26]. Although the Fried criteria and Frailty Index have been widely cited in the literature, these tools are technically complex and time consuming. The FRAIL scale or Simple Frailty Questionnaire (SFQ), comprised of a 5-item, self-reported questionnaire, may be a more practical screening tool [5]. The FRAIL scale is increasingly accepted worldwide and many countries have validated it to their cultural context [5, 27–29].

Few studies have been conducted to assess the validity of frailty diagnosis in Thailand. The Thai Frailty Index (TFI) is constructed according to the accumulated deficits approach and is associated with mortality risk in older people [30]. However, administering the TFI is time consuming. The Simple Frailty Questionnaire could be preferable in busy clinical practices, including preoperative evaluation for elective surgery. Therefore, we performed a cross-cultural adaptation of the questionnaire, assessed its psychometric properties, and explored the optimal cutoff point for frailty in older patients at the surgical preoperative clinic.

## Methods

### Design, setting, and participants

The study aimed to perform a cross-cultural adaptation of the simple frailty questionnaire and assess its validity and reliability in comparison to the Thai Frailty Index in a preoperative setting. This study was conducted in accordance with the Declaration of Helsinki and was approved by Institutional Review Board, Faculty of Medicine Siriraj Hospital, Bangkok, Thailand (COA no. Si 397/2018). This cross sectional-study included 3 hundred patients from the Surgery outpatient clinic at Siriraj Hospital, enrolled from April 2019 to November 2020. Eligible participants were aged 60 years or over and were scheduled for elective surgery. The participants were excluded if they were unable to fluently communicate in Thai. After informed consent was obtained, baseline demographic data were recorded. The TFI and T-FRAIL were collected by two trained research assistants, separately.

### The Thai frailty index (TFI)

The TFI was developed using accumulation deficit model and validated against mortality risk among community dwelling older people [30]. It was created following the suggested standard procedure [31] comprising 30 variables that included medical comorbidities, physical function, cognition and emotional health. The cutoff point of more than 0.25 was used for identifying frailty [30].

### The FRAIL scale and T-FRAIL questionnaires

The FRAIL scale is, 5-item questionnaire for frailty screening. The questionnaire aims to “fatigue”, “resistance”, “ambulation”, “illnesses” and “loss of weight”. Each item required self-report response, which was rated as 1 and 0 points depending on the presence of characteristic in each criterion. The score range from 0 to 5 with higher score demonstrated more frailty characteristics. According to the original FRAIL scale, cutoff point of 3 or above were used to identify frail patient, while score of 2 indicated prefrail status [5]. The T-FRAIL was the Thai version translation of the FRAIL scale which was developed according to the following steps.

### Translation process

Copyright permission for the Simple Frailty Questionnaire translation was granted by the owner, Professor John E. Morley, Division of Geriatric Medicine, Saint Louis University School of Medicine. The guideline for translation is adapted from ‘guidelines for the process of cross-cultural adaptation of self-report measures’ [32].

The translation process included six steps.

#### Step 1 initial translation

The Simple Frailty Questionnaire (FRAIL) was translated from the original English questionnaire by two Thai bilingual independent translators. The first translator was a geriatrician from Siriraj Hospital who was invited to translate the questionnaire from a clinical perspective. The second one was a non-medical translator who translated the questionnaire by language aspect only.

#### Step 2 synthesis of the translations

The research team held the discussion at Siriraj Hospital to review the two Thai versions of the questionnaire in comparison with the original English version. Thai words were carefully chosen from both translations to construct a single version of the questionnaire.

#### Step 3 Back-translation

The Thai version of the questionnaire was sent to two independent language institutions for back-translation. The first translator was invited from the Translation and Interpretation Center, Faculty of Liberal Arts, Mahidol University. The second translator was a Canadian-Thai translator from a private sector organization. These two translators were native English speakers and had no medical background.

#### Step 4 expert committee

An expert committee from Siriraj Hospital was formed to evaluate semantic equivalence, idiom equivalence, experiential equivalence, and conceptual equivalence. The original translation, the Thai questionnaire, and two back-translation versions were included in the discussion.

#### Step 5 test of the prefinal version

A preliminary assessment with 10 elderly participants aged more than 65 years old who attended a geriatric clinic, was done to assess questionnaire comprehension. Most words were easily comprehended by the elders, while some were too difficult. Drawbacks of the current questionnaire were recorded.

#### Step 6 submission of documentation to the developers

The research team adapted and finalized the questionnaire according to the pilot study results. All papers including two back-translations and the finalized Thai questionnaire were sent to the owner of the original version.

### Validity and reliability assessment

#### Content validity

The Thai version of Simple Frailty Questionnaire (T-FRAIL) was evaluated by five independent geriatricians who were not involved in the translation process. Each item of the translated questionnaire was rated in a 4-point scale of ‘1 = not relevant’, ‘2 = somewhat relevant’, ‘3 = quite relevant’ and ‘4 = highly relevant’.

#### Test-retest reliability

The test-retest reliability was conducted in 30 elderly patients on two occasions 7 days apart with the questionnaire items having been rearranged. Later scores were compared with former scores to identify the Intraclass correlation coefficient.

### Concurrent validity and diagnostic test assessment

The concurrent validity including sensitivity, specificity, likelihood ratio for positive and negative results, and the positive and negative predictive value of the T-FRAIL were analyzed in comparison with the TFI as a reference standard. Spearman’s correlation coefficient was estimated to address the correlation between the two tests.

### The T-FRAIL_M1 and the T-FRAIL_M2 questionnaires

The original “illness” criterion in FRAIL scale required 5 or more underlying diseases to count as one score. Patients with several comorbidities were at higher risk of developing complications [33–36] and more likely to receive conservative treatment rather than undergoing surgeries [35–38]. The authors, therefore, anticipated that most patients recruited from preoperative setting for elective surgeries would have number of comorbidities less than 5 and might affect sensitivity of the test. Hence, we proposed T-FRAIL_M1 as a modification of T-FRAIL score by reducing illnesses criterion to 4 or more underlying diseases to determine whether this modification would improve test properties or not, in comparison with the original one. Another anticipated common underreported item would be the loss of weight as it was not a routine measurement among older people in our context. The modification of the questionnaire without this item was initially planned. Moreover, we believe that minimizing the items with reasonable diagnostic properties might facilitate the implementation of the test in busy clinical context. We, therefore, developed T-FRAIL_M2 by exploring the combination of 3 items in T-FRAIL and explore the diagnostic properties of those brief versions.

### Statistical analysis

The content validity index for items (I-CVI) was chosen as the quantification method. Each item’s I-CVI is calculated by the proportion of experts who rated ‘3’ or ‘4’ of all. Excellent content validity required an I-CVI of 1.0 for at least three experts’ analysis [39]. The two-way mixed-effects model with the absolute agreement was used to calculate the intraclass correlation coefficient (ICC) of test-retest reliability. ICC values less than 0.5 were interpreted as poor, between 0.5 and 0.75 as moderate, between 0.75 and 0.9 as good, and greater than 0.90 as excellent [40]. Sensitivity, specificity, likelihood ratio for a positive and negative result, positive predictive value, and negative predictive value were calculated using 2 × 2 tables. Correlation between T-FRAIL and TFI score was demonstrated by Spearman’s correlation coefficient. Each item of the T-FRAIL was evaluated for frailty correlation by the phi correlation coefficient.

## Results

The mean age of the study group (*n* = 300) was 70.4 years (SD 7.3 years), and144 subjects were male (48.0%). Frailty was diagnosed with the TFI in 120 patients (40.0%). Baseline characteristics of the participants are shown in Table 1. The content validity index of items (I-CVI) was 1.0 for all individual items in the T-FRAIL, indicating excellent content validity [39]. Test-retest reliability assessed in 30 patients yielded an intraclass correlation coefficient of 0.880 (95% confidence interval 0.76–0.94) which demonstrates good test-retest reliability [40].

**Table 1:** Characteristics of study population

Patient characteristics (*n* = 300)	n (%)
Age (years), mean ± SD	70.4 ± 7.3
< 70 years	152 (50.7%)
70–79 years	105 (35.0%)
≥ 80 years	43 (14.3%)
Sex	
Male	144 (48.0%)
Female	156 (52.0%)
Education	
Elementary school or lower	193 (64.8%)
High school or above	105 (35.2%)
Body mass index (kg/m^2^)	
Underweight (< 18.5)	20 (6.8%)
Normal (18.5–24.9)	149 (50.3%)
Overweight (25–29.9)	98 (33.1%)
Obese (≥30.0)	29 (9.8%)
Cognitive status	
No cognitive impairment	121 (40.5%)
Cognitive impairment (MoCA< 22/30)	178 (59.5%)
Barthel ADL Index, median (q25,q75)	95 (90,100)
0–20 (suggests total dependence)	2 (0.7%)
21–60 (severe dependence)	15 (5.0%)
61–90 (moderate dependence)	90 (30.0%)
91–100 (slight dependence)	193 (64.3%)
ASA physical status	
Class 1	19 (6.4%)
Class 2	168 (56.2%)
Class 3	105 (35.1%)
Class 4	7 (2.3%)
Underlying disease	
Diabetes mellitus	89 (29.7%)
Hypertension	191 (63.7%)
Cerebrovascular	26 (8.7%)
Chronic kidney disease	66 (22.0%)
Coronary artery disease	44 (14.7%)
Cirrhosis	6 (2.0%)
Cancer	70 (23.3%)
Peripheral vascular disease	6 (2.0%)
COPD	8 (2.7%)
Surgical service	
General surgery	2 (0.7%)
Hepatobiliary surgery	3 (1.0%)
Minimal invasive surgery	11 (3.7%)
Colorectal surgery	7 (2.3%)
Vascular surgery	26 (8.7%)
Urology surgery	40 (13.4%)
Orthopaedics surgery	43 (14.4%)
Endoscopic surgery	102 (34.2%)
Other	64 (21.5%)
Smoking	
Non & Ex-smoker	285 (95.0%)
Current-smoker	15 (5.0%)
Alcohol	
No	275 (91.7%)
Drink	25 (8.3%)
Thai Frailty Index	
Robust	180 (60.0%)
Frail	120 (40.0%)
T-FRAIL	
Fatigue	44 (14.7%)
Resistance	109 (36.3%)
Ambulation	102 (34.0%)
Illness	21 (7.0%)
Loss of weight	93 (31.0%)

Abbreviations: *MoCA* The Montreal Cognitive Assessment; *ADL* Activities of Daily Living, *ASA* the American Society of Anesthesiologists, *COPD* Chronic obstructive pulmonary disease, *T-FRAIL* The Thai version of the Simple Frailty Questionnaire

T-FRAIL yielded an excellent accuracy against TFI, with an area under the receiving operating characteristic curve of 0.882. A strong correlation between T-FRAIL and TFI was represented by Spearman’s correlation coefficient of 0.708 (*p* = < 0.001). The cutoff-point for T- FRAIL of two points showed the highest Youden index of 63.1% compared to 43.3% of the original cutoff score of three. (Table 2) The cutoff of two points had a sensitivity of 77.5% (95% CI 69.0–84.6) and a specificity of 86.1% (95%CI 79.6–90.3), which was a better sensitivity than at three points. The positive predictive value of the two-point cutoff was 78.2% (95%CI 71.2–83.8), while the negative predictive value was 85.1% (95% CI 80.3–88.9).

**Table 2:** Frailty cut-point for original T-FRAIL with TFI as the reference standard

Frailty cut-points	Number of frail patients (%)	Sensitivity (%)	Specificity (%)	Youden’s Index (%)	PPV (%)	NPV (%)
≥ 1	182 (60.7%)	93.3	61.1	54.4	61.5	93.2
≥ 2	119 (39.7%)	77.5	85.6	63.1	78.2	85.1
≥ 3	57 (19.0%)	45.0	98.3	43.3	94.7	72.8
≥ 4	10 (3.3%)	8.3	100	8.3	100	62.1

Abbreviations: *T- FRAIL* The Thai version of the Simple Frailty Questionnaire, *TFI* The Thai Frailty Index, *PPV* positive predictive value, *NPV* negative predictive value

Elderly individuals with high comorbidities might be excluded from elective surgery due to the risks of the procedure. To explore its diagnostic properties, we modified the T-FRAIL by reducing the threshold of the “illnesses” criterion. A modification that reduced comorbid diseases from five to four (T-FRAIL_M1) and applied a cutoff point score of two improved test parameters (Table 3). Sensitivity was 83.3% (95% CI 75.4–89.5) and specificity was 82.8 (95% CI 76.5–88.0), which is much better than the original T-FRAIL at a cutoff point of three.

**Table 3:** Frailty cut-point for T-FRAIL_M1 with TFI as the reference standard

Frailty cut-points	Number of frail patients (%)	Sensitivity (%)	Specificity (%)	Youden’s Index (%)	PPV (%)	NPV (%)
≥ 1	189 (63.0%)	94.2	57.8	52	59.8	93.7
≥ 2	131 (43.7%)	83.3	82.8	66.1	76.3	88.2
≥ 3	69 (23.0%)	53.3	97.2	50.5	92.8	75.8
≥ 4	26 (8.7%)	20.8	99.4	20.2	96.2	65.3

Abbreviations: *T-FRAIL_M1* modified T-FRAIL by reducing “illness” criterion to 4 or more diseases

“Resistance” and “ambulation” were the best T-FRAIL items to identify frailty. The “resistance” yielded a sensitivity of 72.5% and specificity of 87.8%, while the “ambulation” yielded a sensitivity of 68.3% and specificity of 88.9% (Table 4). Another proposed modification of the T-FRAIL, each questionnaire item was combined into sets of three components (T-FRAIL_M2). A positive response from at least one item out of the three was considered as frail. The “fatigue”, “resistance”, and “illnesses” combination showed the highest Youden’s index of 66.4% with a sensitivity of 85.8% and a specificity of 80.6%. All modified questionnaires with three items were analyzed for sensitivity and specificity in comparison with the TFI. (Table 5).

**Table 4:** Sensitivity, specificity, and Youden’s index of each original T-FRAIL item

	TFI		SFQ Items		
	Robust (*n* = 180)	FRAIL (*n* = 120)	Sensitivity	Specificity	Youden’s index
Fatigue	11 (6.1%)	33 (27.5%)	27.5%	93.9%	21.4
Resistance	22 (12.2%)	87 (72.5%)	72.5%	87.8%	60.3
Ambulation	20 (11.1%)	82 (68.3%)	68.3%	88.9%	57.2%
Illnesses	4 (2.2%)	18 (15.0%)	15.0%	97.8%	12.8%
Loss of weight	43 (23.9%)	50 (41.7%)	41.7%	76.1%	17.8

Abbreviations: *T- FRAIL* The Thai version of the Simple Frailty Questionnaire, *TFI* The Thai Frailty Index, *SFQ Items* The Simple Frailty Questionnaire items

**Table 5:** Sensitivity, specificity and Youden’s index in T-FRAIL_M2

	Combination of SFQ Items		
	Sensitivity (%)	Specificity (%)	Youden’s index (%)
1, 2, 3; positive at least 1 item	83.3%	78.3%	61.6
1, 2, 4; positive at least 1 item	85.8%	80.6%	66.4
1, 2, 5; positive at least 1 item	90.8%	64.4%	55.2
1, 3, 4; positive at least 1 item	79.2%	80.6%	59.8
1, 3, 5; positive at least 1 item	85.8%	65.6%	51.4
1, 4, 5; positive at least 1 item	66.7%	71.7%	38.4
2, 3, 4; positive at least 1 item	84.2%	81.1%	65.3
2, 3, 5; positive at least 1 item	88.3%	64.4%	52.7
2, 4, 5; positive at least 1 item	89.2%	65.6%	54.8
3, 4, 5; positive at least 1 item	85.0%	67.8%	52.8

Note: 1 represents “Fatigue”; 2 represents “Resistance”; 3 represents “Ambulation”; 4 represents “Illnesses”; 5 represents “Loss of Weight”

Abbreviations: *T-FRAIL_M2* modified T-FRAIL by the combination of three components with positive results if presence any feature from three parameters, *SFQ Items* The Simple Frailty Questionnaire items

## Discussion

The T-FRAIL was translated with a standardized protocol and achieved satisfying psychometric properties, including good or excellent content validity and test-retest reliability. The questionnaire was tested against the TFI and achieved excellent accuracy. Previous validity studies of the simple frailty questionnaire in other contexts have yielded varying results due to different methodologies and different populations.

The original FRAIL scale, using positive at three out of five item criteria, showed a significant correlation with impaired function in a cross sectional cohort [5], but the study did not investigate the correlation with the reference standard or using a different cutoff point. Nevertheless, several studies using various cutoff points were identified among later cross-culturally adapted versions of the FRAIL scale [27, 28, 41]. For example, using three points to diagnose frailty, the Korean (K-FRAIL) was tested against a comprehensive geriatric assessment-based frailty index as a reference standard and demonstrated excellent (90%) sensitivity but low (33%) specificity [27]. Meanwhile, the FRAIL-AR, using similar 3-point cutoff score, was compared with Fried Frailty Index with sensitivity and specificity of 72 and 67%, respectively [41]. Using a three-point cutoff score, we observed a significantly lower sensitivity with very high specificity, which may not be optimal for a screening tool.

Nevertheless, some studies in Chinese and Japanese populations (FRAIL-J) using a 2-point cutoff have yielded comparable results. In the Chinese study, A 2-point cutoff Chinese FRAIL scale represented a sensitivity of 90% and a specificity of 86% compared to the Fried Frailty Phenotype [28]. Similarly, the FRAIL-J was compared to Fried Frailty phenotype with a sensitivity of 77.8% and specificity of 88.9% [42]. Our modification using the original T-FRAIL at a cutoff score of two showed similar properties to the Japanese version. However, further modification by reducing the number of medical illnesses (T-FRAIL_M1), improves sensitivity from 77 to 83% with a better Youden’s index at 66%. Moreover, further modification of the cutoff point to be as low as one point for both the original T-FRAIL and T-FRAIL_M1 would yield a higher sensitivity of 93 and 94%, respectively, with a specificity of around 60%. This provides better properties compared to the 3-point score of K-FRAIL [27].

Another proposed method to modify the T-FRAIL modification is to lower the criterion of the questionnaire. According to the Study of Osteoporotic Fractures index (the SOF index) for frailty diagnosis, weight loss, inability to rise from a chair, and poor energy are sufficient to classify pre-frail and frail patients in comparison with the Fried Frailty phenotype. The SOF index also proved the relationship to clinical outcomes including falls, disability, fracture, and mortality in two large studies [43, 44]. We also modified the T-FRAIL instrument to improve its psychometric properties using a combination of three components (T-FRAIL_M2), resulting in a sensitivity and specificity of around 80%.

Although the T-FRAIL_M1 and the T-FRAIL_M2 improved sensitivity of the test, overall test properties were similar to the T-FRAIL. The AuROC of T-FRAIL_M1 and T-FRAIL_M2 were around 83%, while the original one was 81%. Despite its little difference, the modification leads to more sensitivity which serves author’s propose to use the questionnaire for screening test rather than confirmation test. Moreover, the T-FRAIL_M2, due to its simplicity, would be useful for primary care setting in rural area, where many citizens had lower education level. According to the Table 5, despite omitting the “loss of weight” criteria, the T-FRAIL_M2 (“fatigue”, “resistance” and “illnesses”) still had high performance for frailty diagnosing. This fact emphasized the T-FRAIL_M2 advantages in Thai context, whose most elders could not recall their previous bodyweight.

An optimal pre-operative screening tool should have high sensitivity and an acceptable specificity. The cutoff score of one point from five items from the original version of T-FRAIL and T-FRAIL_M1 would serve this purpose. Nevertheless, in resource limited settings, an alternative option could be the T-FRAIL_M1 with a 2-point cutoff score or T-FRAIL_M2 that provides reasonable sensitivity and better specificity.

The strengths of our study were the use of reference standard in addition to the standard translational procedure. The reference standard in this study was derived from large scale population in our country and has been studied for the validity in the local setting. Nevertheless, additional studies of predictive validity in stratifying risk in the elderly would be valuable. Further study to identify the elderly who might experience adverse outcomes or benefit from specific interventions using this test are needed. Limitation of the study was in relation to the studying in one setting in single center. All of our subjects were selected from elective surgery, the generalizability of the findings to other settings might be an issue. Additional studies among older people in similar setting at other center and in other medical context would ascertain the usability of the test.

## Conclusion

The T-FRAIL demonstrated good validity and reliability for diagnosing frailty in older people preparing for elective surgery. A modified T-FRAIL using a lower cutoff point yielded higher sensitivity than the original cutoff point but gave lower specificity. Another modification was a reduction of the illnesses criterion or a combination with sets of three components increased sensitivity, while preserved excellent specificity of the test, making it suitable for use as a screening tool in practical use. A prospective study is needed to determine if the T-FRAIL and its modified versions may be useful to predict mortality, disability, or other clinical outcomes in different settings.

## Data Availability

The datasets used and/or analyzed during the current study are available from the corresponding author on reasonable request.

## Abbreviations

T- FRAIL: Thai version of the Simple Frailty Questionnaire
T-FRAIL_M1: Modified T-FRAIL by reducing “illnesses” criteria to 4 or more diseases
T-FRAIL_M2: Modified T-FRAIL by combination with sets of three components
TFI: Thai Frailty Index
I-CVI: Content validity index for items
FRAIL: Simple Frailty Questionnaire
ICC: Intraclass correlation coefficient
K-FRAIL: Korean version of the FRAIL scale
FRAIL-AR: Arabic version of the FRAIL scale
FRAIL-J: Japanese version of the FRAIL scale
SOF: Study of Osteoporotic Fractures
